# Expanded Spectrum and Increased Incidence of Adverse Events Linked to COVID-19 Genetic Vaccines: New Concepts on Prophylactic Immuno-Gene Therapy, Iatrogenic Orphan Disease, and Platform-Inherent Challenges

**DOI:** 10.3390/pharmaceutics17040450

**Published:** 2025-03-31

**Authors:** Janos Szebeni

**Affiliations:** 1 Nanomedicine Research and Education Center, Department of Translational Medicine, Semmelweis University, 1089 Budapest, Hungary; jszebeni2@gmail.com; 2 SeroScience LCC, 1125 Budapest, Hungary; 3 Translational Nanobioscience Research Center, Sungkyunkwan University, Suwon 16419, Republic of Korea

**Keywords:** LNP, lipid nanoparticle, mRNA, Comirnaty, Spikevax, vaccinations, side effects, gene therapy, immunotherapy, COVID-19 pandemics, Brighton list

## Abstract

The mRNA- and DNA-based “genetic” COVID-19 vaccines can induce a broad range of adverse events (AEs), with statistics showing significant variation depending on the timing and data analysis methods used. Focusing only on lipid-nanoparticle-enclosed mRNA (mRNA-LNP) vaccines, this review traces the evolution of statistical conclusions on the prevalence and incidence of AEs associated with these vaccines, from initial underestimation of atypical, severe toxicities to recent claims suggesting the possible contribution of COVID-19 vaccinations to the excess deaths observed in many countries over the past few years. Among hundreds of different AEs listed in Pfizer’s pharmacovigilance survey, the present analysis categorizes the main symptoms according to organ systems, with nearly all of them being affected. Using data from the US Vaccine Adverse Event Reporting System and a global vaccination dataset, a comparison of the prevalence and incidence rates of AEs induced by genetic versus flu vaccines revealed an average 26-fold increase in AEs with the use of genetic vaccines. The difference is especially pronounced in the case of severe ‘Brighton-listed’ AEs, which are also observed in COVID-19 and post-COVID conditions. Among these, the increases in incidence rates relative to flu vaccines, given as x-fold rises, were 636x, 530x, 220x, 231x, 155x, 90x, and 133x for myocarditis, thrombosis, death, myocardial infarction, tachycardia, dyspnea, and hypertension, respectively. This review delineates the concept that genetic vaccines can be regarded as prophylactic immuno-gene therapies and that the observed chronic disabling AEs might be categorized as iatrogenic orphan diseases. It also examines the unique vaccine characteristics that could be causally related to abnormal immune responses, which potentially lead to adverse events and complications. These new insights may contribute to improving the safety of this platform technology and assessing the risk/benefit balance of various products.

## 1. Introduction

mRNA-based vaccines, Pfizer-BioNTech’s BNT162b2 (Comirnaty) and Moderna’s mRNA-1273 (Spikevax), became the most widely used preventive measure against the SARS-CoV-2 virus during the COVID-19 pandemic. Through the blending of nanotechnology with genetic engineering, this innovative approach introduced a novel class of medical intervention with promising applications beyond vaccination [1,2,3].

However, like any groundbreaking technology, this innovation has brought along challenges, such as the unexpected rise of a broad range of adverse events (AEs) and complications. This was not a major issue until the gravity and lethality of the COVID-19 pandemic made the overwhelming benefit of vaccination over the risk of the disease unambiguous. Later on, however, as a result of the success of global immunization campaigns and gradual attenuation of the pathogenicity of new virus variants, the World Health Organization declared (in May 2023) that COVID-19 was no longer a global public health emergency. At this point, the risk/benefit ratio should have been reevaluated in light of the increasing number of severe, disabling AEs [4,5]. Nevertheless, sustaining COVID-19 immunity through repeated vaccinations remains the prevailing suggestion of guidelines, as demonstrated by the US FDA’s recent approval of updated mRNA vaccines for adults and children, including ’emergency use’ authorization for infants aged 6 months and older [6]. The AEs and complications associated with these vaccines remain unresolved, necessitating a reassessment of the risk/benefit ratio of mRNA vaccinations. A better understanding of the issue’s scope and the mechanisms driving these AEs would be instrumental in this assessment. This review seeks to offer such an analysis.

Although the AE profile of DNA-based genetic vaccines, such as AstraZeneca’s Vaxzevria and Johnson & Johnson/Janssen’s Jcovden, may in some regards be even worse than that of the mRNA vaccines, these vaccines have been withdrawn from the market and are therefore not included in the present review.

## 2. The “Special Interest” Symptoms of Post-Vaccination Syndrome and Public Reaction

The collection of AEs caused by the COVID-19 mRNA vaccines has been called “post-vaccination syndrome” [7,8,9,10], and some of these symptoms overlap with those of COVID-19 and post-COVID, referred to as “symptoms of special interest” or “Brighton case” symptoms, a compilation of AEs by the “Brighton Collaboration”, an international network of experts in drug and vaccine safety [11,12,13,14]. Over the past few years, post-vaccination syndrome has attracted widespread public and scientific attention. Concerns have been raised that these AEs and complications may have played a role in the excess mortality recorded in various Western nations in recent years [15,16]. Physician coalitions have called for a moratorium, legal actions for compensation have been filed, and political debates have reached major institutions such as the British Parliament and the U.S. Congress. Additionally, public hearings and media discussions continue to showcase insights from experts, vaccine-injured individuals, and concerned public figures. Analysis of the literature in PubMed [17], using the search engine End-Note (and search terms “COVID-19”, “mRNA vaccines”, and “adverse events”) returned nearly 1500 articles (January, 2025) focusing on AEs and challenging the universal claim that these vaccines are “safe”. The latter statement is based on the low incidence rate of AEs in the 0.03–0.5 % range (see later), defined as the number of AE reactors related to the overall number of vaccine recipients in a certain time-window. Indeed, the above incidence range counts as low by pharmacotherapy standards, where higher AE rates are generally accepted. However, vaccines differ in this regard, as AEs in a large population of healthy individuals are less acceptable than in patients receiving pharmacotherapy for existing illnesses. Additionally, the global scale of these vaccinations has led to a very high prevalence of AEs, i.e., the total number of affected people in a certain time, imposing a significant burden on society. For these reasons, the accurate quantification of vaccine-induced AEs is critical to assessing their risk/benefit ratio. Unfortunately, in the case of COVID-19 vaccines, the AE statistics vary significantly based on the time, data collection, and analysis methods.

## 3. The Unique Features of mRNA Vaccines and Their Adverse Effects

The mRNA in the Comirnaty and Spikevax codes for de novo, in loco antigen synthesis in immune cells represents a revolutionary innovation in vaccine technology. Its advantages include the simplification, acceleration, and cost-reduction of vaccine production [18]. This efficiency facilitates a quick response to viral mutations and allows for the possibility of delivering multiple antigens at once, enabling combined vaccines against multiple viral strains.

Table 1 shows an organ system-classified list of COVID-19 adverse events of special interest (AESIs), which is used to identify those special vaccine-induced severe AEs that resemble COVID-19, rather than those caused by traditional vaccines. The spectrum of AEs is uniquely broad and includes rare symptoms and diseases that are atypical for most other vaccines, drugs, or even toxic agents, but which are common in infection with SARS-CoV-2. This points to one or more very fundamental mechanisms of interference with multiple biological processes that are also seen in COVID-19 and post-COVID-19 syndrome. Obviously, the occasional manifestation of AEs must depend on individual genetic and epigenetic factors, just as the rise and spectrum of symptoms in acute and chronic (long) COVID-19.

## 4. Prevalence and Incidence of Adverse Events Caused by mRNA-LNP Vaccines: Inconsistent Statistics

Clinical trials conducted before approval, large-scale post-marketing safety surveillance, prospective multicenter studies, and AE monitoring systems have yielded significantly different statistics on the AEs caused by Comirnaty.

In the initial phase II/III randomized clinical trial studying the safety, tolerability, immunogenicity, and efficacy of RNA vaccine candidates against COVID-19 in healthy individuals (ClinicalTrials.gov ID: NCT04368728) 21,720 and 21,728 subjects were vaccinated with Comirnaty or placebo, respectively. Polack et al. reported no significant difference between the vaccine and placebo groups in the incidence of mild, common side effects of vaccinations. The observed severe AEs were claimed to have a “low incidence” in both groups that were similar to those caused by other viral vaccines [19]. This was the pivotal study leading to the emergency use authorization of Comirnaty. However, a secondary analysis of the same data by Fraiman et al., counting the Brighton-listed AEs [12], found a 36% higher risk of severe AEs in the vaccine group compared to placebo. As it turned out, the selection of AEs for statistical analysis was limited only to the mild symptoms in the Polack et al. study [19], while the reanalysis focused on severe, Brighton-case AEs. The statistics in the latter study showed 18 (1.2–34.9, 95% CI) serious AEs over placebo in 10,000 participants, corresponding to 1 person displaying a severe vaccine-induced AE in about 556 participants (0.18%) [12]. The ratio of “special interest” AEs among all serious AEs was ~56% [12].

Three months after the global rollout of Comirnaty, Pfizer-BioNTech’s originally confidential, now publicly accessible, post-authorization safety report [20] gave an account of 42,086 AE case reports on 158,893 events out of 126,212,580 vaccine doses in 56 countries. This means that 0.13% and 0.03% AE incidences were related to events or reactors, respectively, or one reactor for every ~3000 vaccine recipients. Reactions were observed mainly in the 31–50-year-old age range and three -times more often in women than man, and full recovery ensued in 47%. The rest recovered with sequalae or did not recover within 3 months. The report listed 2.9% fatality among the reactors (1223 deaths), implying ~0.001% of overall vaccinations resulted in fatality, or one death in about one hundred thousand (103,200) vaccinations. However, the relationship between vaccination and reported death is uncertain.

Beyond the fact that 53% of the reactive individuals recovered with sequalae or did not recover within 3 months, what is astonishing in this report is the approximately 1,100 different words or terms for AEs used in the appended nine-page cumulative list of AEs [20]. Among many unique, unprecedented AEs, the list contained ~220 different types of conditions including the syllable “itis” (implying inflammation) and/or the word “autoimmune” (which is an inflammatory process). Thus, about 20% of all AEs involves inflammation.. Nevertheless, the surveys’ summary aligned with the conclusion of the Phase II-III study [19], claiming that “the data do not reveal any novel safety concerns or risks requiring label changes”. 

The statement on safety was reinforced in an international 6-month efficacy and safety study involving 21,926 recipients of Comirnaty and an equal number of participants who received placebo [21]. This study reported a 16.3% occurrence of “any event” over placebo, which is about 125 times higher than the AE event incidence in Pfizer’s 3-month safety surveillance (0.13%) [20], and the 0.51% of severe reactions was about 100-fold higher than that in the 3-month safety surveillance study [20]. Nevertheless, again, “no new safety signals were identified during the longer follow-up period” [21].

The next set of comprehensive statistics, provided by the Centers for Disease Control and Prevention (CDC) and the Food and Drug Administration (FDA), were based on continuous monitoring through various reporting systems, including the Vaccine Adverse Event Reporting System (VAERS) [22,23]. Unlike clinical trials wherein the documentation of AEs is part of the study, the VAERS entries are volunteer reports by healthcare providers and patients. The first 6-month post-marketing analysis of the AEs attributed to Comirnaty and Spikevax, based on the VAERS data [24,25], gave a 0.11% AE reactor incidence rate (out of 298,792,852 doses), near four-fold higher than the corresponding value in the 3-month Pfizer survey (0.03%) [20].

One reason for the above ambiguities regarding the occurrence of mRNA-LNP-induced AEs is inconsistency in data collection. A 2010 study by the U.S. Department of Health and Human Services estimated that fewer than 1% of vaccine AEs and only 1–13% of serious events are reported to VAERS [26]. The reporting process appears to be complex, and not all AEs are documented, especially if they are mild or if the individual does not associate them with vaccination.. Despite these limitations, for comparing AEs associated with COVID-19 vaccines with those associated with other vaccines, such as flu vaccines, VAERS appeared to be the most suitable data source.

## 5. Comparison of mRNA-LNP and Flu Vaccines

In addition to the prevalence and incidence of AEs, which reflect on the clinical impact of side effects, another key question regarding vaccine safety is how the risk of AEs compares to that of other vaccines, especially those that are also offered to a large population. In the case of COVID-19 mRNA vaccines, seasonal flu vaccines may serve as the best reference since they are also administered to millions and target an airborne virus, like SARS-CoV-2. Table 2 compares the prevalence and incidence of AEs associated with Comirnaty, Spikevax, and Jcovden (Janssen), a DNA-containing vaccine developed by Johnson & Johnson, with those associated with flu vaccines. The latter data were obtained by aggregating the AEs of 12 flu vaccines listed in VAERS (see legend to Table 2).

It is seen in the table that the incidence rate of AEs caused by the analyzed COVID-19 vaccines over 2.5 years was 25–26 times higher than that of flu vaccines during the same time. Considering only the DNA-based vaccine, Jcovden, the relative risk of experiencing an AE compared to the flu vaccine is 54-fold higher. This also means that the DNA vaccine caused ~2-fold more AEs than the mRNA vaccines. A comparison of Comirnaty and Spikevax suggested 57% more reactions in the case of Spikevax. The substantial difference between the flu vaccine and the 2 mRNA vaccines and the relative similarity between Comirnaty and Spikevax in causing AEs provide clear indication that it is the mRNA-LNP technology, rather than any other special features of the 2 mRNA vaccines, that accounts for the increased risk of AEs. On the other hand, the 20- and 32-fold increase in relative risk calculated for Comirnaty vs. Spikevax shows comparably increased toxicities, for which that of Spikevax is somewhat higher than that of Comirnaty.

Statistics on individual AEs: Using the flu vaccines as a comparator, Table 3 shows the incidence rates of 12 Brighton-case AEs caused by the mRNA and flu vaccines in order of decreasing prevalence.

As in the case of all AEs combined (Table 2), the mRNA-LNP vaccine-induced incidence rates of all 12 distinct AEs were massively higher than those after flu vaccination, with heart disease and thrombosis having the highest, roughly a ~1200- and ~500-fold increased risk, respectively. These data also show that the incidence rates of different AEs substantially vary within the 6–200 AEs/M range.

The vaccine-related fatal outcomes reported to VAERS (Table 3) suggest approximately one death per 33,000 vaccine recipients, or an incidence rate of ~0.003%, which is close to the ~0.001% reported in Comirnaty’s 3-month post-market surveillance [20]. These ratios, taken together with the immunization statistics (a total of 4600 M Comirnaty and 817 M Spikevax doses distributed across the world through 2024), imply death cases approximately in the 55,000–160,000 range worldwide. However, as mentioned, the death reports are ambiguous as they could be coincidental, usually associated with a comorbidity. A further useful piece of information is that the percent of severe AEs relative to all AEs is in the ~4 and ~18% range [24,28,29], although in the 3-month post-market surveillance [20] it reached or exceeded 40–50% for many AEs.

**Figure 1:**
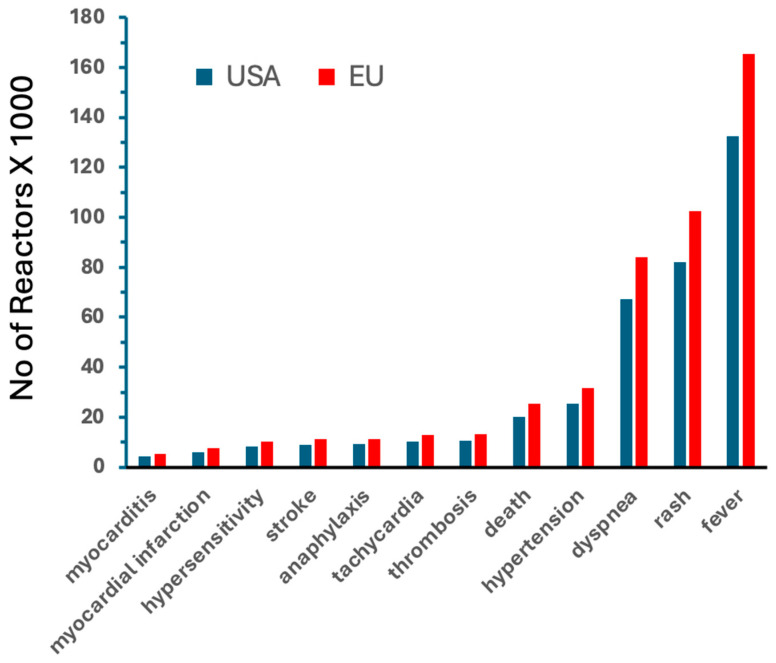
Rough estimates of the prevalence of mRNA vaccine-induced AEs in the USA and Europe during the COVID-19 pandemic, between December 2020 and May 2023. The calculations of the total number of AE reactors were based on the incidence rates for mRNA vaccines shown in Table 3 (obtained from the VAERS), which were multiplied by the number of Comirnaty + Spikevax mRNA doses injected during this period, obtained from the Our World in Data public database [27].

Figure 1 shows rough estimates of the prevalence of mRNA vaccine-induced different AEs only in the USA and Europe during the COVID-19 pandemic, based on dose statistics and VAERS-based incidence rates in the USA (italicized as *AE/M* in column 5 of Table 3). The increasing order of bars visualizes the conclusion, that because of the larger number of vaccinations in Europe, the prevalence of different symptoms is also higher in Europe. It is also notable in the figure that after fever, rash and dyspnea were the most frequent AEs, whose coincidental occurrence is typical of liposome and other nanoparticle-induced complement (C) activation [30,31,32], as discussed below in detail.

## 6. Complement Activation as a Possible Contributor to Acute AEs

Most attention paid to vaccine AEs is focused on the inflammatory and autoimmune complications that affect the heart, nerve, and coagulation systems, overlooking the fact noted in Figure 1 that the -runner-up symptoms in the AE prevalence list are rash, and dyspnea. These may be due to pseudoallergy that results from complement activation by the LNPs.. Thus, the association of skin symptoms (e.g., rash, urticaria) with cardiopulmonary distress, which manifests as dyspnea, hyper- and hypotension, tachycardia, bradycardia, or arrhythmia, suggests the involvement of complement activation [30,31,32]. Two other AEs that could also be related to complement activation are thrombosis and thrombocytopenia, since anaphylatoxins and the terminal complement complex activate the endothelial cells and platelets [33,34,35]. Further support for the involvement of complement activation in the observed acute vaccine reactions is the fact that the “AEs of special interest”, associated with mRNA vaccines, share similarities with the inflammatory symptoms of COVID-19, wherein intense complement activation plays a key pathogenic role [36,37,38,39,40]. Furthermore, C3, the central molecule in the complement activation cascade, is a ubiquitous innate mediator of inflammatory responses undergoing activation in most systemic inflammatory processes [41]. Nevertheless, the strongest and most direct evidence for complement activation playing a role in the observed acute AEs is that Comirnaty is a potent complement activator, as shown in pigs [42], human serum in vitro [43], and pig blood in vivo [44].

As for the mechanism of complement activation, we observed the involvement of the alternative pathway in human serum [43], while, in sera containing high anti-PEG antibody titers, complement activation via the classical pathway also becomes prominent [44]. As pointed out earlier, virtually all components of Comirnaty have the capability to activate complement, including the ionizable lipid (ALC-0315), DSPC, cholesterol, and the spike protein [32].

In conclusion, the most immediate and frequent inflammatory symptoms associated with mRNA vaccines may be due, in a great part, to complement activation. The produced anaphylatoxins, in addition to causing anaphylactic reactivity [45], also “spark the flame in early autoimmunity” [46,47]. The hypersensitivity reaction caused by complement activation, called complement activation-related pseudoallergy (CARPA) [30,31,32], can be life threatening in people with high anti-PEG antibody level and/or severe allergy [46]. Hence, genetic predisposition to allergic and/or autoimmune reactions, such as atopic constitution, represents a risk factor for vaccine-induced acute hypersensitivity reactions, anaphylaxis, or autoimmunity. Importantly, these reactions can be prevented or attenuated by complement inhibitors [41,48,49].

**Figure 2:**
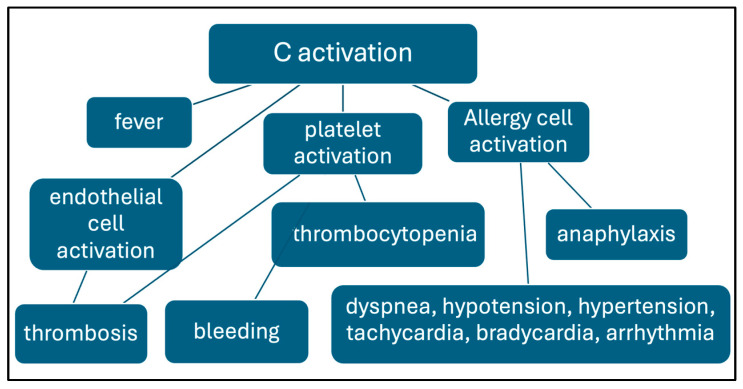
Reaction sequence of complement-mediated AEs caused by mRNA vaccines. The connections between the framed processes and symptoms suggest causal relationships.

Because of the resemblance to pathogenic viruses, complement activation by the vaccine nanoparticles, an inherent feature of LNPs [32], is biologically rationalizable. Thus, beyond the vaccines, other present and future products of the mRNA-LNP technology may have to face the complement activation problem. Figure 2 outlines the reaction sequence by which vaccine-induced complement activation may cause the discussed AEs.

## 7. Regulatory Classification of mRNA Vaccines

Despite the wide acceptance of the use of “mRNA vaccines”, the regulatory classification of Comirnaty and Spikevax is ambiguous. The proposed classification terms include “biotechnological medicines”, “biological products/drugs/medicines”, and “gene-based vaccines”. Some authors even argue that these formulations are not even vaccines but representations of gene therapy, as they exert the transfection of genetic material (nucleic acid) to change genetic information. However, it may not be considered as gene therapy on the grounds that the DNA does not change, at least in theory, and that the therapeutic goal of vaccination is not genetic correction. A possible alternative to all above terminologies is immuno-gene therapy, a term that has previously been applied in cancer immunotherapy [50]. It expresses that gene translation is used to modify an immune phenomenon, namely, to accelerate the processing and presentation of an antigen. Its implications for public health and regulatory standards lie in the more accurate definition of the intervention.

## 8. The Orphan Disease Proposition for Categorizing Persistent and/or Disabling Vaccine-Induced Chronic AEs

An orphan disease is a rare medical condition that affects a small percentage of the population and therefore lacks sufficient research, treatment options, and financial incentives for drug development [51,52,53,54]. These diseases are usually genetic disorders, such as Huntington’s disease, cystic fibrosis, Duchenne muscular dystrophy, and certain rare cancers.

Despite the higher incidence of vaccine-related AEs compared to flu vaccines (Table 2 and Table 3), Figure 1 shows that the cumulative number of mRNA vaccine-induced chronic, potentially persistent, debilitating AEs (myocarditis, myocardial infarction, stroke, thrombosis), as of May 2023, remained below about 10,000 in the U.S., which is well below the threshold of 200,000 patients used to define the upper limit for orphan disease categorization in the U.S. [53]. These vaccine injuries, in addition to being orphan diseases, could also be identified as “iatrogenic”, acknowledging that, unlike the genetic orphan diseases, they are unintended consequences of a medical intervention. The significance of this distinction lies in the fact that many countries have implemented special policies for orphan diseases that ensure dedicated research funding and the development of specialized treatments [52,53,54]. The U.S. Orphan Drug Act of 1983 [55] provides a relevant example of initiatives aimed at addressing the needs of patients with rare diseases.

## 9. The European Experience: Paul-Ehrlich-Institute Statistics

The COVID-19 vaccine-induced AEs are closely monitored in Europe as well. In Germany, the Paul Ehrlich Institute (PEI), a participant in the WHO-led Vaccine Safety Net project, serves as a primary source of statistics on genetic vaccine-induced AEs [28]. According to the PEI, the incidence rate of severe AEs (of special interest) associated with mRNA vaccines was approximately 0.2 per 1000 doses, or 0.02% [28]. For comparison, the corresponding values from various U.S. statistics mentioned earlier in this review were 0.03% [20], 0.13% (VAERS, Table 2), 0.18% [12], and 0.5% [21].

Table 4 presents the incidence rates of AEs caused by mRNA- and DNA-containing vaccines, published by PEI [28]. While the list and rank of symptoms differ from the US data (Table 3), cardiopulmonary distress (e.g., dyspnea, arrhythmia) is among the top on the AE list with both types of vaccines. As discussed above, these symptoms can be linked to activation of the complement system, providing additional support for the causal role of complement activation in the lead AEs of genetic vaccines. 

**Figure 3:**
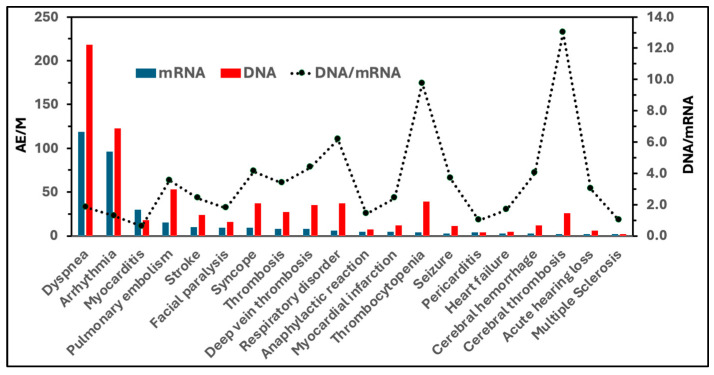
Incidence rates of “special interest” AEs caused by mRNA and DNA-based genetic vaccines in Germany (visual representation of data from Table 4). Blue bars represent the summed incidences (AEs/M) associated with mRNA vaccines (Comirnaty and Spikevax), while red bars represent those associated with DNA vaccines (Jcovden and Vaxzevria). The dotted line (right y-axis) shows the DNA/mRNA ratios, indicating the fold-increase in the incidence of each AE observed with DNA vaccines compared to mRNA vaccines.

Figure 3 compares the incidence rates of DNA vaccine-induced AEs and those associated with mRNA vaccines. The bars with different colors represent the combined AE/M values for the 2 mRNA (Comirnaty and Spikevax) and 2 DNA (Vaxzevria and Jcovden) vaccines, and the secondary y-axis (dotted line) quantitates the ratio of AE incidence rates between the two vaccine types. Except for myocarditis and pericarditis, these rates were at least twice as high for the DNA vaccines compared to the mRNA vaccines. Notably, DNA vaccines showed a 10–13-fold higher incidence of thrombocytopenia and cerebral thrombosis, which aligns with their suspension and eventual withdrawal due to thrombotic thrombocytopenia and severe cerebral events, particularly sinus thrombosis. As previously noted, complement activation plays a key pathogenic role in these hemostatic abnormalities [33,35], which is in keeping with the fact that adenoviruses -used in DNA vaccines-, are particularly strong activators of the complement system [56].

## 10. Potential Plausible Causes of Adverse Events Inherent to the mRNA-LNP Platform

**Figure 4:**
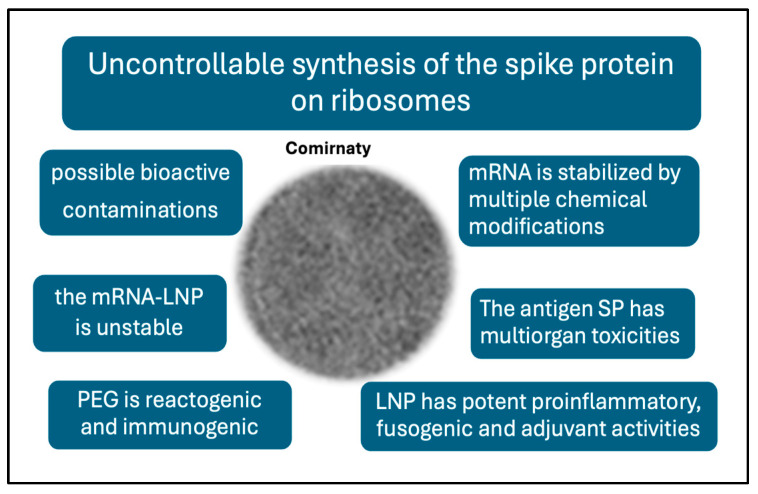
Unique features of the mRNA vaccines that are not characteristic of traditional vaccines and may be linked to AEs.

It seems likely that the root cause of vaccine-induced AEs and complications may be the same as the main concern with gene therapy, namely unintended immune processes, off-target effects, and unforeseen toxicities [57]. It was mentioned that complement activation is an inherent property of mRNA-LNPs; however, the idea map in Figure 4 suggests many more features of mRNA vaccines that are different from those of traditional vaccines, which may be linked to AEs.

These differences include the following [58]: (i) The highly controlled, multistep pathway of antigen processing and presentation in natural immunogenicity is replaced in mRNA vaccines by uncontrollable ribosomal synthesis of the antigen. This results in a diversification of SP processing and presentation in unnatural ways [58]. (ii) Nucleoside and other chemical modifications increase the stability of the synthetic mRNA, and, hence, the efficacy of SP translation and its in vivo stability [59,60]. (iii) The prefusion-stabilized antigen SP has diverse, multiorgan toxicities [61,62,63]. Its rapid entry into the bloodstream and whole-body tissue distribution [64,65] can cause autoimmune reactions and a variety of cell and tissue damages. (iv) The LNP is a robust proinflammatory agent [66,67] and multiorgan mRNA transfectant [68,69] which underlies systemic inflammation and autoimmune phenomena. (iv) The stabilizer polymer (polyethylene glycol, PEG) on the vaccine nanoparticle surface has anaphylactic reactogenicity and immunogenicity, implying possible anti-vaccine immune reactivity [44,46]. (v) the vaccine nanoparticles are unstable in water [70]. (vi) The injectable vaccine may contain residual DNA fragments, [71], odd molecular assemblies, and inorganic metals and complexes [72]. The above deviations from traditional vaccines offer plausible explanations for the AEs of mRNA vaccines [58], which may be perceived as collateral damage from the otherwise successful fight against COVID-19. 

**Figure 5:**
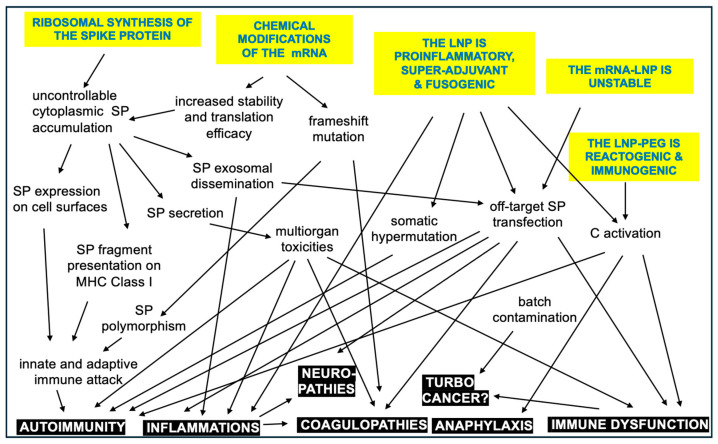
Hypothetic immune processes and phenomena contributing to the AEs caused by mRNA vaccines. The arrows point from cause to potential effect in the intertwined reaction cascade. The inherent vaccine properties are itemized with blue in yellow background, and the adverse events or complications are white in black. It needs to be strongly emphasized that this diagram is NOT a reaction scheme of the normal operation of the vaccine in non-reactive people [73].

Figure 4 presents a spider map of possible adverse vaccine features, a comprehensive theory on the root causes of AEs. The concept is expanded into a flowchart of potential cause–effect relationships among the reactions and symptoms (Figure 5), wherein the suggested steps may occur independently or simultaneously and may act in additive or synergistic ways. Furthermore, the emergence and severity of AEs are influenced by genetic and epigenetic factors, as well as various external and internal conditions.

## 11. Outlook

The rare occurrence of severe AEs during mass vaccinations is not unprecedented. A notable example is the 1976 swine flu pandemic in the U.S., where an increase in the incidence of AEs (e.g., Guillain–Barré syndrome) and complications resulted in approximately 30 deaths after 43 M vaccinations, ultimately leading to the suspension of the vaccine [74]. This example underscores that the term “safe” is relative and depends on varying criteria across different times and contexts.

At present, the mainstream scientific literature and public health institutions hold the mRNA-LNP vaccines safe with a high benefit/risk ratio. Accordingly, this technology platform is garnering unprecedented interest and investment. The FDA has recently approved Moderna’s second mRNA vaccine, mRESVIA (mRNA-1345), against respiratory syncytial virus [75,76], and over 300 new mRNA-LNP-based drugs are in development across dozens of companies. Novel mRNA vaccines targeting influenza, Zika virus, HIV, cytomegalovirus, and cancer are undergoing clinical trials, and a great number of preclinical studies suggest the potential utility of mRNA-LNPs as anticancer immunotherapies and multivalent vaccines [77,78,79]. However, the AEs discussed in this review present a biological barrier to the success of the mRNA-LNP technology. Therefore, as the field advances, it is crucial to develop a deeper understanding of the immune mechanisms underlying the AEs and complications potentially caused by genetic vaccines and other mRNA-LNP-based products.

## Figures and Tables

**Table 1:** List of COVID-19 adverse events of special interest (AESIs) *.

Organ System	Adverse Events
**Cardiovascular**	acute coronary syndrome, aneurysm, arrhythmia, arrhythmias, arteriosclerosis, cardiac tamponade, coronary artery disease, deep vein thrombosis, cardiomyopathy, edema of the lip, tongue, face, endothelial dysfunction, heart failure, hypertension, hypotension, ischemia, large-vessel vasculitis, microangiopathy, myocardial infarction, myocarditis, non-bacterial thrombotic endocarditis, pericarditis, postural tachycardia syndrome, stroke, sudden death, Takotsubo syndrome (stress cardiomyopathy), vascular inflammation (Kawasaki disease)
**Neurological**	acute disseminated encephalomyelitis, ageusia, anosmia, aseptic meningitis, Bell’s palsy, cerebral venous sinus thrombosis, CNS bleed, chronic fatigue syndrome, cranial polyneuropathy, dysesthesia with exanthem, dysgeusia, encephalitis, encephalopathy, facial nerve palsy, Guillain-Barré syndrome, hypogeusia, hypoglossal nerve palsy, hyposmia, myalgic encephalomyelitis, myoclonus, myoclonus-ataxia, ophthalmoplegia, oropharyngeal dysphagia, persistent hiccups, seizures, sensorineural hearing loss, stroke (hemorrhagic, ischemic), vestibular neuritis
**Respiratory**	acute chest syndrome, acute respiratory distress syndrome, bronchospasm, bullous lung disease, coughing, dyspnea, pulmonary vasculitis, hemopneumothorax, hemoptysis, hilar lymphadenopathy, hoarseness, hypoxia, pediatric croup, platypnea orthodeoxia syndrome, pneumomediastinum, pneumothorax, pneumomediastinum, pulmonary embolism, stridor
**Gastrointestinal**	acute acalculous cholecystitis, angular cheilitis, appendicitis, cholecystitis, colitis, enteritis, enterocolitis, fulminant hepatic failure, hepatitis, intussusception, pancreatitis, paralytic ileus, parotitis, spontaneous hemoperitoneum, spontaneous splenic rupture, tongue ulcers
**Musculoskeletal**	arthralgia, arthritis, aseptic arthritis, muscle spasms, myalgia, myositis, rhabdomyolysis
**Dermatological**	chilblain, chronic urticaria, cutaneous vasculitis, dermatographia, dermatomyositis, epidermal necrolysis, erythema multiforme, follicular eruption, Gianotti-Crosti rash, Gilbert type erythema nodosum, Grover-like eruption, hyperkeratosis, lower extremity bullae, maculopapular rash, nail bed red half-moon sign, oral vesiculobullous lesions, painful cystic lesion, perniosis-like lesions ("COVID toes"), pityriasis rosea, pustular eruption, rash, seborrheic dermatitis, small fiber neuropathy, Steven-Johnson syndrome, unilateral thoracic exanthema, urticaria (hives), vasculitis.
**Hematological**	anemia, coagulopathy, cold agglutinin syndrome, hemophagocytic lymph histiocytosis, idiopathic thrombocytopenic purpura, lymphopenia, methemoglobinemia, stroke, thromboembolism, thrombocytopenia, thrombosis, thrombosis with thrombocytopenia
**Endocrine/** **Metabolic**	adrenal injury, diabetes, hyperglycemia, myxedema, orchitis, pancreatitis, parotitis, prostatitis, prostatic infarction, sexual dysfunction, thyroiditis
**Renal/** **Genitourinary**	glomerulopathy, hematuria, hypernatremia, IgA vasculitis with nephritis, nephrosis, proteinuria, renal failure, renal infarction, urinary retention, vasculitis with glomerulonephritis
**Immune System**	anaphylaxis, autoimmune flare-ups, autoimmune glomerulonephritis, autoimmune hemolytic anemia, autoimmune hepatitis, autoimmune rheumatological diseases, CARPA, hypersensitivity reactions, lymphadenopathy, multiorgan autoimmunity, multiorgan inflammation, persistent lymph-node enlargement, swelling and pain, peripheral lymphoedema formation and pain
**General/Systemic**	abscess, alopecia, hyperferritinemic syndrome, hyperglycemia, hyponatremia, multisystem inflammatory syndrome, sepsis, septic shock
**Psychiatric**	akathisia, altered mental status, catalepsy, convulsions, delirium, insomnia, mania, multiple sclerosis, narcolepsy, psychosis, seizures, status epilepticus, sudden and persistent dysphonia, suicide attempt
**Ocular**	bilateral macular bleed, bilateral visual loss, conjunctivitis, episcleritis, ocular myasthenia gravis, ocular/orbital inflammation, retinopathy, uveo-retinitis
**Reproductive**	abortion, ectopic pregnancy, fetal HELLP syndrome (hemolysis, elevated liver enzymes, and low platelet count)
**Gynecological/** **obstetric**	amenorrhea, dysmenorrhea, endometritis, irregular bleeding, menorrhagia, metrorrhagia, multiple uterine myomatosis, oligomenorrhea, pelvic inflammatory disease, postmenopausal recurrent bleeding, premature bleeding, premenstrual syndrome
**Oncological**	acute lymphocytosis, lymphoid leukemias, “turbo cancer”

* Among other sources, data from the “Safety Platform for Emergency vaccines SO2-D2.1.2 Priority List of COVID-19 Adverse events of special interest: Quarterly update December 2020, https://brightoncollaboration.org/wp-content/uploads/2023/08/SO2_D2.1.2_V1.2_COVID-19_AESI-update_V1.3-1.pdf, accessed on 23 March 2025. Bold entries are disease categories or organ systems.

**Table 2:** VAERS-reported adverse events associated with genetic (mRNA and DNA) COVID-19 vaccines and 12 flu vaccines combined, from December 2020 to May 2023.

Vaccine	AE+ n *	Doses	AE+/M **	AE−/AE+ ***	COVID/Flu
Comirnaty	434,821	401,685,954	1082	924	20
Spikevax	426,714	251,852,502	1694	590	32
Combined mRNA	861,535	653,538,456	1318	759	25
Jcovden (Janssen)	54,728	18,991,177	2882	347	54
All genetic	934,959	672,529,633	1390	719	26
Flu	18,696	352,670,000	53	18,863	1

AE + n *, total number of individuals reporting one or more AEs within 1 day after vaccination, regardless of severity. AE/M **, AEs per million vaccine doses; AE-/AE+ ***, proportion of nonreactors relative to reactors; COVID-19/Flu, genetic vaccine/flu vaccine AE/M ratio. The administered vaccine doses were “Word in Data” information [27]. The flu vaccines included in the statistics comprised various tri- or quadrivalent products with the brand names: AFLURIA (CSL-Limited, Parkville, Melbourne, Australia and Seqirus Inc., Maidenhead, UK), FLUAD (Novartis, Basel, Switzerland and Seqirus Inc.), FLUARIX (GlaxoSmithKline, GSK, Brentford, London, UK), FLUBLOK (Protein Sciences Corp., Meriden, CN, USA), FLUCELVAX (Novartis, Seqirus Inc.), FLUENZ TETRA (Medimmune Vaccines, Gaithersburg, MD, US), FLULAVAL (GSK), FLUMIST (Medimmune Vaccines), and FLUZONE (Sanofi Pasteur, Lyon, France). Three other flu vaccines considered had no brand names.

**Table 3:** VAERS data on the prevalence and incidence of selected flu and COVID-19 mRNA vaccine-induced AEs in the US from December 2020 to May 2023.

	Flu Vaccines		mRNA Vaccines		Fold Increase	
	AE	AE/M	AE	*AE/M*	AE	AE/M
fever	4294	7.9	132,447	*201.70*	31	26
rash	1118	2.06	82,113	*125.05*	73	61
dyspnea	622	1.14	67,355	*102.57*	204	90
hypertension	160	0.29	25,292	*38.52*	158	133
death	74	0.14	20,227	*30.8*	273	220
thrombosis	19	0.03	10,439	*15.9*	549	530
tachycardia	52	0.1	10,205	*15.54*	196	155
anaphylaxis	117	0.22	9094	*13.85*	78	63
stroke	280	0.52	8939	*13.61*	32	26
hypersensitivity	122	0.22	8153	*12.42*	67	56
myocardial infarction	23	0.04	6067	*9.24*	264	231
myocarditis	3	0.01	4176	*6.36*	1392	636

The data collection method and abbreviations are similar to those in Table 2, except that the analysis performed in SQL (Structured Query Language) was searching for multiple synonyms for each symptom, to make sure that they were counted as one. The exact cause of death is not specified in VAERS. The data in column 4 (AE/M) are listed in order of decreasing incidence rate and are italicized to highlight that they were also used for calculating the AE prevalences in Figure 1. Other conditions are the same as in Table 2.

**Table 4:** The incidence rates of “special interest” AEs* caused by two mRNA and two DNA-containing genetic vaccines in Germany.

AE of Special Interest	AEs/Million			
	Comirnaty	Spikevax	Vaxzevria	Jcovden
Dyspnea	55	64	110	108
Arrhythmia	46	50	57	66
Myocarditis	14	16	6	12
Pulmonary embolism	8	7	33	20
Stroke	6	4	15	9
Facial paralysis	5	4	7	9
Syncope	5	4	25	12
Thrombosis	4	4	19	8
Deep vein thrombosis	4	4	27	8
Respiratory disorder	3	3	33	4
Anaphylactic reaction	3	2	4	3
Myocardial infarction	3	2	6	6
Thrombocytopenia	3	1	32	7
Seizure	2	1	7	4
Pericarditis	2	2	1	3
Heart failure	2	1	2	3
Cerebral hemorrhage	2	1	8	4
Cerebral thrombosis	1	1	20	6
Acute hearing loss	1	1	5	1
Multiple Sclerosis	1	1	1	1

* Data collected by the Paul Erlich Institute between 2 December 2020 and March 2022 [28]. Vaxzevria and Jcovden are the brand names of the COVID-19 vaccines developed by AstraZeneca/Oxford University and Janssen (Johnson & Johnson), respectively.
