# Correction: Szebeni, J. Expanded Spectrum and Increased Incidence of Adverse Events Linked to COVID-19 Genetic Vaccines: New Concepts on Prophylactic Immuno-Gene Therapy, Iatrogenic Orphan Disease, and Platform-Inherent Challenges. *Pharmaceutics* 2025, *17*, 450

**DOI:** 10.3390/pharmaceutics17070801

**Published:** 2025-06-20

**Authors:** Janos Szebeni

**Affiliations:** 1 Nanomedicine Research and Education Center, Department of Translational Medicine, Semmelweis University, 1089 Budapest, Hungary; jszebeni2@gmail.com; 2 SeroScience LCC, 1125 Budapest, Hungary; 3 Translational Nanobioscience Research Center, Sungkyunkwan University, Suwon 16419, Republic of Korea

To make the sentences clearer and easier for readers to read, the grammatical format of some sentences has been modified, as follows:

1. Change “on the prevalence of AEs and incidents” into “on the prevalence and incidence of AEs ” and change “The review” into “This review” in abstract.
2. Change “visualize” into “visualizes” in Section 5.
3. Change “Similar data collection and abbreviations as” into “The data collection method and abbreviations are similar to those”, change “was done” into “performed”, change “using AE symptom search of” into “was searching for”, change “making” into “to make”, change “italicized for highlighting that they are” into “italicized to highlight that they were” in the footnote of Table 3.
4. Change “activation.s” into “activation”, change “an ubiquitous” into “a ubiquitous” in Section 6.
5. Add “diverse” after “SP has”, change “multiorgan distribution” into “whole-body tissue distribution”, change “multisystem” into “multiorgan”, add “systemic” after “underlies”, delete “from the otherwise successful fight against COVID-19” in Section 10.
6. Change “Mrna” into “mRNA” in References [7,12,21,32,59,63,70].

**Error in Abstract and Table**

In the original publication [1], there was a mistake in Table 3: some of the AE/M values in the seventh column, obtained by dividing the AE/M for mRNA vaccines (fifth column) by those for flu vaccines (third column), were slightly inaccurate due to inadvertent division errors. The corrected values, also quoted in the Abstract (line 14)—”Among these, the increases in incidence rates relative to flu vaccines, given as x-fold rises, were 636x, 530x, 220x, 231x, 155x, 90x, and 133x for myocarditis, thrombosis, death, myocardial infarction, tachycardia, dyspnea, and hypertension, respectively”—do not affect the scientific conclusions of the review. However, the correction attenuates the magnitude of the mRNA-vaccine-induced myocarditis incidence increase relative to flu vaccines by approximately a factor of 2, still representing the highest, 636-fold-elevated risk for myocarditis.

**Abstract:** The mRNA- and DNA-based “genetic” COVID-19 vaccines can induce a broad range of adverse events (AEs), with statistics showing significant variation depending on the timing and data analysis methods used. Focusing only on lipid-nanoparticle-enclosed mRNA (mRNA-LNP) vaccines, this review traces the evolution of statistical conclusions on the prevalence and incidence of AEs associated with these vaccines, from initial underestimation of atypical, severe toxicities to recent claims suggesting the possible contribution of COVID-19 vaccinations to the excess deaths observed in many countries over the past few years. Among hundreds of different AEs listed in Pfizer’s pharmacovigilance survey, the present analysis categorizes the main symptoms according to organ systems, with nearly all of them being affected. Using data from the US Vaccine Adverse Event Reporting System and a global vaccination dataset, a comparison of the prevalence and incidence rates of AEs induced by genetic versus flu vaccines revealed an average 26-fold increase in AEs with the use of genetic vaccines. The difference is especially pronounced in the case of severe ‘Brighton-listed’ AEs, which are also observed in COVID-19 and post-COVID conditions. Among these, the increases in incidence rates relative to flu vaccines, given as x-fold rises, were 636x, 530x, 220x, 231x, 155x, 90x, and 133x for myocarditis, thrombosis, death, myocardial infarction, tachycardia, dyspnea, and hypertension, respectively. This review delineates the concept that genetic vaccines can be regarded as prophylactic immuno-gene therapies and that the observed chronic disabling AEs might be categorized as iatrogenic orphan diseases. It also examines the unique vaccine characteristics that could be causally related to abnormal immune responses, which potentially lead to adverse events and complications. These new insights may contribute to improving the safety of this platform technology and assessing the risk/benefit balance of various products.

**Table 3:** VAERS data on the prevalence and incidence of selected flu and COVID-19 mRNA vaccine-induced AEs in the US from December 2020 to May 2023.

	Flu Vaccines		mRNA Vaccines		Fold Increase	
	AE	AE/M	AE	*AE/M*	AE	AE/M
fever	4294	7.9	132,447	*201.70*	31	26
rash	1118	2.06	82,113	*125.05*	73	61
dyspnea	622	1.14	67,355	*102.57*	204	90
hypertension	160	0.29	25,292	*38.52*	158	133
death	74	0.14	20,227	*30.8*	273	220
thrombosis	19	0.03	10,439	*15.9*	549	530
tachycardia	52	0.1	10,205	*15.54*	196	155
anaphylaxis	117	0.22	9094	*13.85*	78	63
stroke	280	0.52	8939	*13.61*	32	26
hypersensitivity	122	0.22	8153	*12.42*	67	56
myocardial infarction	23	0.04	6067	*9.24*	264	231
myocarditis	3	0.01	4176	*6.36*	1392	636

The data collection method and abbreviations are similar to those in Table 2, except that the analysis performed in SQL (Structured Query Language) was searching for multiple synonyms for each symptom, to make sure that they were counted as one. The exact cause of death is not specified in VAERS. The data in column 4 (AE/M) are listed in order of decreasing incidence rate and are italicized to highlight that they were also used for calculating the AE prevalences in Figure 1. Other conditions are the same as in Table 2.

The authors state that they did not significantly alter the fundamental content and conclusions. This correction was approved by the Academic Editor, and the original publication has been updated accordingly.
